# Sepsisversorgung in Deutschland: Qualitätsindikatoren zu Diagnostik und Therapie der Sepsis

**DOI:** 10.1007/s00101-026-01663-5

**Published:** 2026-03-19

**Authors:** Christian S. Scheer, Evgeny A. Idelevich, Simon Oelsner, Jacob Packmohr, Konrad Reinhart, Matthias Gründling

**Affiliations:** 1 Klinik für Anästhesiologie, Intensiv‑, Notfall- und Schmerzmedizin, Universitätsmedizin Greifswald, Ferdinand-Sauerbruch-Straße 17475, Greifswald, Deutschland; 2 Friedrich Loeffler-Institut für Medizinische Mikrobiologie, Universitätsmedizin Greifswald, Greifswald, Deutschland; 3 Institut für Medizinische Mikrobiologie, Universitätsklinikum Münster, Münster, Deutschland; 4 Klinik für Anästhesiologe und Operative Intensivmedizin, Charité Universitätsmedizin Berlin, Berlin, Deutschland

**Keywords:** Sepsis, Qualitätsmanagement, Qualitätsindikatoren, IQTIG, Initiative zur Qualitätsverbesserung, Sepsis, Quality management, Quality indicators, IQTIG, Quality improvement initiative

## Abstract

**Hintergrund:**

In Deutschland wurden 2026 Qualitätsindikatoren des Instituts für Qualitätssicherung und Transparenz im Gesundheitswesen (IQTIG) zu Diagnostik und Therapie der Sepsis in Krankenhäusern verpflichtend eingeführt. Bisher existieren kaum Daten, inwiefern Qualitätsindikatoren in Form von Maßnahmen zu Sepsiserkennung und -therapie bereits etabliert sind.

**Methode:**

Sekundäranalyse des European Sepsis Care Survey, einer internationalen Querschnittstudie zu Strukturen der Sepsisversorgung in Akutkrankenhäusern. In Deutschland wurden leitende Ärzte aus Krankenhäusern aller Bundesländer und aller Versorgungsstufen systematisch zur Teilnahme eingeladen. Die Analyse erfolgte in Bezug auf 7 Qualitätsindikatoren des IQTIG zu Diagnostik und Therapie der Sepsis.

**Ergebnisse:**

In Deutschland wurden 253 Krankenhäuser (20 % aller Akutkrankenhäuser) analysiert. Ein standardisiertes Screening zur Früherkennung einer Sepsis existierte krankenhausweit in 34,8 % (95%-KI:27,9–42,3 %), standardisierte Maßnahmen zum Sepsismanagement in 36,6 % (95%-KI 29,2–44,5 %) der Krankenhäuser. Dabei bestanden Unterschiede zwischen Notaufnahmen, Normalstationen und Intensivstationen (*p* < 0,05), aber keine signifikanten Unterschiede zwischen universitären und nichtuniversitären Krankenhäusern. Regelmäßige Schulungen des ärztlichen und pflegerischen Dienstes aller Abteilungen existierten in 4,7 % (95%-KI:2,3–8,5 %) der Krankenhäuser.

**Schlussfolgerung:**

In Deutschland existierten krankenhausweite standardisierte Maßnahmen zu Früherkennung und Behandlung einer Sepsis nur in wenigen Krankenhäusern. Regelmäßige Schulungen des ärztlichen und pflegerischen Personals zur Qualitätsverbesserung wurden kaum durchgeführt. Vor diesem Hintergrund ist die Etablierung von qualitätsverbessernden Programmen dringend geboten, bedarf aber gleichzeitig klarer Strukturen und ausreichender Ressourcen.

**Graphic abstract:**

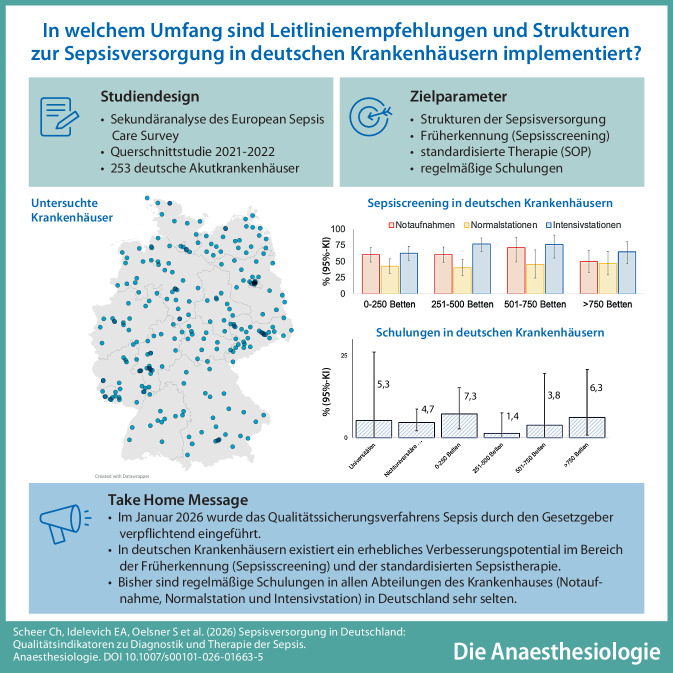

**Zusatzmaterial online:**

Die Online-Version dieses Beitrags (10.1007/s00101-026-01663-5) enthält die zugrunde liegende Umfrage. Bitte scannen Sie den QR-Code.

## Abstract (en)

**Background:**

In Germany quality indicators from the Institute for Quality Assurance and Transparency in the Healthcare System (IQTIG) on the diagnostics and treatment of sepsis in hospitals will be compulsorily introduced in 2026. Currently, there are few data on the extent to which quality indicators in the form of measures to recognize and treat sepsis are already established.

**Methods:**

This was a secondary analysis of the European Sepsis Care Survey, an international, cross-sectional study of the organization of sepsis care in acute care hospitals. Head physicians from hospitals in all federal states and at all levels of care in Germany were systematically invited to participate. The analysis investigated seven quality indicators of the IQTIG on the diagnostics and treatment of sepsis.

**Results:**

In Germany 253 hospitals (20% of all acute care hospitals in Germany) were analyzed. Standardized screening for early detection of sepsis existed in 34.8% (95% confidence interval, CI 27.9–42.3%) of hospitals and standardized measures for sepsis management in 36.6% (95% CI 29.2–44.5%) of hospitals. There were differences between emergency departments, general wards and intensive care units (*p* < 0.05) but no significant differences between university and non-university hospitals. Regular training of medical and nursing staff in all departments existed in 4.7% (95% CI 2.3–8.5%) of hospitals.

**Conclusion:**

In Germany only a few hospitals had hospital-wide standardized measures for the early detection and treatment of sepsis. Regular training of medical and nursing staff to improve quality was rarely implemented. Against this background, the establishment of quality improvement programs is urgently needed but at the same time requires clear structures and sufficient resources.

## Hintergrund

Sepsis gehört zu den häufigsten Notfällen in Deutschland. Die Krankenhaussterblichkeit ist mit über 40 % hoch [1–3]. Hinzukommen eine hohe Morbidität und Letalität nach überlebter Sepsis [4]. Die Kosten der Sepsisbehandlungen im Krankenhaus werden in Deutschland auf 11,7 Mrd. € jährlich geschätzt [5]. Eine schnelle, strukturierte, zielgerichtete und damit qualitativ hochwertige Therapie ist daher entscheidend, um die Krankheitsschwere zu reduzieren, die Sterblichkeit zu senken und die oft schwerwiegenden Langzeitfolgen, die bei 75 % der Überlebenden auftreten, zu reduzieren [2].

Seit vielen Jahren existieren nationale und internationale Leitlinien, die neben einem standardisierten Screening zur Früherkennung, evidenzbasierte Maßnahmen zur Behandlung sowie krankenhausweite Schulungen und Trainings empfehlen [6, 7]. Die Weltgesundheitsorganisation (WHO) hat 2017 die Bedeutung eines umfassenden Sepsismanagements in ihrer Resolution 70.7 betont [8]. In Europa wurde das European Centre for Disease Prevention and Control (ECDC) kürzlich von mehreren internationalen Fachgesellschaften aufgerufen, Sepsis als Thema aufzunehmen [9].

In Deutschland wurde 2020 das Institut für Qualitätssicherung und Transparenz im Gesundheitswesen (IQTIG) durch den Gemeinsamen Bundesausschuss (G-BA) mit der Entwicklung eines Qualitätssicherungsverfahrens „Diagnostik, Therapie und Nachsorge der Sepsis“ für Krankenhäuser beauftragt [10, 11]. Im Ergebnis wird die systematische Erhebung von Qualitätsindikatoren im Jahr 2026 durch den Gesetzgeber verpflichtend eingeführt. Diese neuen Qualitätsindikatoren, die durch ein Expertengremium entwickelt wurden, beziehen sich auf wesentliche Maßnahmen in der Sepsisversorgung und sind in ihrem Umfang stark begrenzt [12–15]. Ziel des Qualitätssicherungsverfahrens Sepsis ist, eine Reduzierung der Letalität und neu auftretender Morbidität zu erreichen. Darüber hinaus soll das zukünftige Qualitätssicherungsverfahren (QS) geeignet sein, die Prozess‑, Struktur- und Ergebnisqualität der stationären Leistungserbringung, inklusive der Notaufnahmen/Rettungsstellen, bei der Behandlung erwachsener Patientinnen und Patienten mit Sepsis einrichtungsvergleichend abzubilden [13–15].

Bisher existieren kaum Daten, in welchem Umfang Qualitätsmaßnahmen zu Sepsiserkennung, mikrobiologischer Diagnostik, strukturierter Behandlung, antiinfektiver Therapie, Antibiotic Stewardship, regelmäßigen Schulungen sowie Erhebung der Sepsissterblichkeit und anderer Qualitätsparameter in deutschen Krankenhäusern bereits existieren.

## Methode

Im Rahmen einer Sekundäranalyse wurden Daten des European Sepsis Care Survey (ESCS) [16] zu Krankenhausstrukturen und Prozessen der Sepsisversorgung in deutschen Krankenhäusern der Akutversorgung untersucht. Das European Sepsis Care Survey wurde von der European Sepsis Alliance initiiert und durch mehrere internationale wissenschaftliche Fachgesellschaften (European Society of Anaesthesiology and Intensive Care [ESAIC], European Society of Intensive Care Medicine [ESICM], European Society of Emergency Medicine [EUSEM], European Society of Clinical Microbiology and Infectious Diseases [ESCMID], Intensive Care Society United Kingdom [ICS UK], European society for paediatric intensive care, paediatric critical care & neonatal intensive care [ESPNIC], European Shock Society [ESS], International Fluid Academy [IFA]) unterstützt. Die Datenerfassung des ESCS erfolgte von August 2021 bis August 2022.

In Deutschland waren Krankenhäuser einschlussfähig, die über Abteilungen wie Notaufnahmen, Innere Medizin, Chirurgie, Neurologie und/oder Intensivmedizin verfügten. In diesen Krankenhäusern wurden im Rahmen einer systematischen Vollerhebung telefonisch bzw. per E‑Mail die Leitenden der Notaufnahmen, der Intensivstationen oder des Qualitätsmanagements kontaktiert. Die Teilnahme erfolgte online anhand eines Fragebogens (Zusatzmaterial online). Für den European Sepsis Care Survey existieren eine Zustimmung der Ethikkommission der Universitätsmedizin Greifswald (BB 124/21) und eine Registrierung unter clinicaltrials.gov (Identifier: NCT05059808).

Für diese Subgruppenanalyse wurden die Ergebnisse der deutschen ESCS-Teilnehmer im Hinblick auf die vom IQTIG beschlossenen Qualitätsindikatoren des Qualitätssicherungsverfahrens Sepsis ([13–15]; Tab. 1) untersucht.

**Tab. 1:** Qualitätsaspekte und Qualitätsindikatoren des Qualitätssicherungsverfahrens (QS) Sepsis des IQTIG sowie des European Sepsis Care Survey zur Sepsisversorgung in Krankenhäusern.

	Qualitätssicherungsverfahrens Sepsis des IQTIG [15]	European Sepsis Care Survey [16]
*Qualitätsaspekt*	*Qualitätsindikator zur Strukturqualität*	
*Infektionspräventive Maßnahmen zur Vermeidung von Sepsis*	Multimodales Präventionsprogramm von zentralvenösen Gefäßkatheter-assoziierten Infektionen zur Prävention von Sepsis im Krankenhaus (einrichtungsbezogene Dokumentation)	*Nicht erfasst*
*Schulungen der Gesundheitsprofessionen zu Diagnostik und Therapie der Sepsis*	Regelmäßige Schulungen zu Erkennung, Risikoeinstufung und Therapie von Sepsis (einrichtungsbezogene Dokumentation)	Regelmäßiges Sepsistraining oder ein Programm zur Qualitätsverbesserung bei Sepsis (Schulungen von Ärzten und Pflegekräften, in Notaufnahme, auf Stationen und Intensivstationen) (einrichtungsbezogene Analyse)
*Antiinfektive Therapie der Sepsis*	Therapieleitlinie zur antiinfektiven Therapie unterstützt durch ein multidisziplinäres Antibiotic Stewardship Team (einrichtungsbezogene Dokumentation)	SOP oder Leitlinie für die antimikrobielle Behandlung von Patienten mit Sepsis (einrichtungsbezogene Analyse)
		Antibiotic Stewardship Teams (einrichtungsbezogene Analyse)
*Standardisierte Prozesse zu Diagnostik und Therapie von Patientinnen und Patienten mit Sepsis*	Arbeitsanweisung (SOP) zur Versorgung bei Sepsis (einrichtungsbezogene Dokumentation)	Protokoll, Behandlungspfad oder Bundle zur Behandlung einer Sepsis (einrichtungsbezogene Analyse)
*Qualitätsindikator zur Prozessqualität*		
*Einstufung des Sepsisrisikos*	Screening mittels Messinstrumenten zur Einstufung des Sepsisrisikos (fallbezogene Dokumentation)	Standardisiertes Screening zur Erkennung von Sepsis (in Notaufnahme, auf Normalstation und auf der Intensivstation) (einrichtungsbezogene Analyse)
*Durchführung einer mikrobiologischen Diagnostik*	Blutkulturen vor Beginn der antimikrobiellen Therapie der Sepsis (fallbezogene Dokumentation)	Blutkulturen vor Beginn einer antiinfektiven Therapie (einrichtungsbezogene Analyse)
*Qualitätsindikator zur Ergebnisqualität*		
*Outcome*	Krankenhaus-Letalität nach Sepsis/neu aufgetretene Morbidität (fallbezogene Dokumentation)	Systematische Registrierung der Sepsissterblichkeit (einrichtungsbezogene Analyse)

Die Tabelle stellt die Qualitätsaspekte und Qualitätsindikatoren des QS Sepsis den im European Sepsis Care Survey untersuchten Aspekten der Versorgungsqualität gegenüber. Im QS Sepsis werden zukünftig Qualitätsindikatoren zur Strukturqualität anhand der einrichtungsbezogenen Dokumentation und Qualitätsindikatoren zu Prozess- und Ergebnisqualität anhand der fallbezogenen Dokumentation überprüft. Im Gegensatz dazu basieren die im European Sepsis Care Survey erfassten Daten auf einer einrichtungsbezogenen Analyse, die anhand eines Fragebogens erfolgte.

Dazu wurden die Elemente des ESCS, die Qualitätsindikatoren zu Struktur‑, Prozess und Ergebnisqualität in der Sepsisversorgung darstellen, analysiert (Tab. 1). Die im European Sepsis Care Survey erfassten Daten basieren dabei auf einer einrichtungsbezogenen Analyse, die anhand eines Fragebogens (Zusatzmaterial online) erfolgte. Im Gegensatz dazu werden im QS Sepsis Qualitätsindikatoren zur Strukturqualität zukünftig anhand der einrichtungsbezogenen Dokumentation und Qualitätsindikatoren zu Prozess- und Ergebnisqualität anhand der fallbezogenen Dokumentation überprüft.

Für ein detaillierteres Bild wurden universitäre und nichtuniversitäre Krankenhäuser unterschieden und die Krankenhäuser entsprechend ihrer Größe in vier Strata unterteilt (Stratum 1: 0 bis 250 Betten, Stratum 2: 251 bis 500 Betten, Stratum 3: 501 bis 750 Betten und Stratum 4: > 750 Betten). In den Analysen wurden nur definitive Antworten (ja/nein, eindeutig benanntes Kriterium oder Maßnahme) berücksichtigt. Die Ergebnisse wurden als absolute Zahlen und prozentuale Anteile mit 95 %-Konfidenzintervallen (95%-KI) dargestellt. *p*-Werte wurden anhand des Chi-Quadrat-Tests berechnet (Signifikanzlevel α < 0,05).

## Ergebnisse

**Abb. 1:**
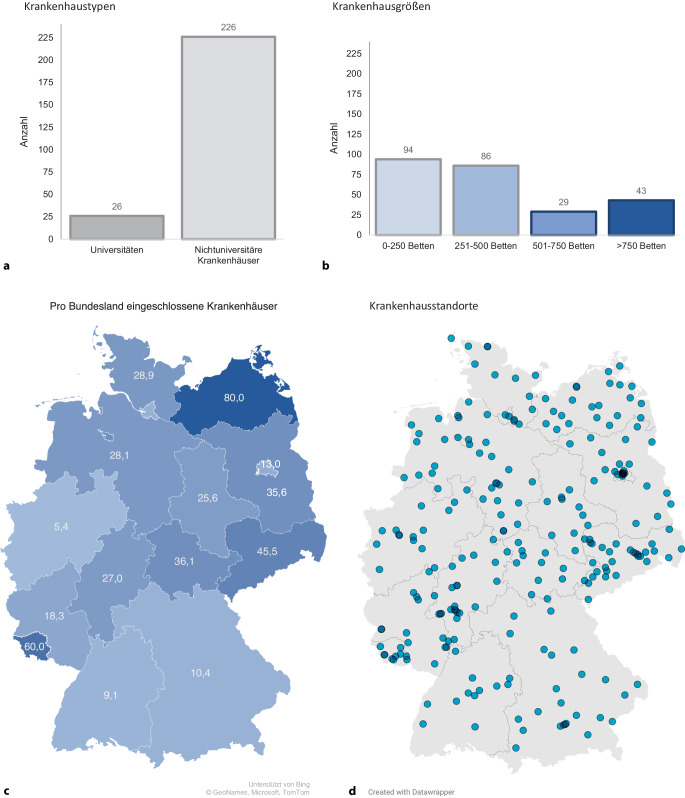
Eingeschlossene Krankenhäuser **a** nach Krankenhaustyp; **b** nach Krankenhausgröße; **c** nach Bundesland und **d** Krankenhaustandorte

Insgesamt konnten Informationen aus 253 deutschen Krankenhäusern aus allen 16 Bundesländern analysiert werden. Eingeschlossen wurden Krankenhäuser aller Größen (65 bis 3300 Betten), in ländlichen und städtischen Regionen, Kreiskrankenhäuser, Regionalversorger und universitäre Kliniken (Abb. 1). Die Fragen des ESCS wurden in 63 % durch Einrichtungsleitungen und in 23 % durch Oberärzte und Oberärztinnen, vor allem aus den Bereichen Notfallmedizin, Intensivmedizin, Anästhesiologie, Innere Medizin und Chirurgie, beantwortet.

### Sepsiserkennung, standardisiertes Management und Schulungen des Personals

**Abb. 2:**
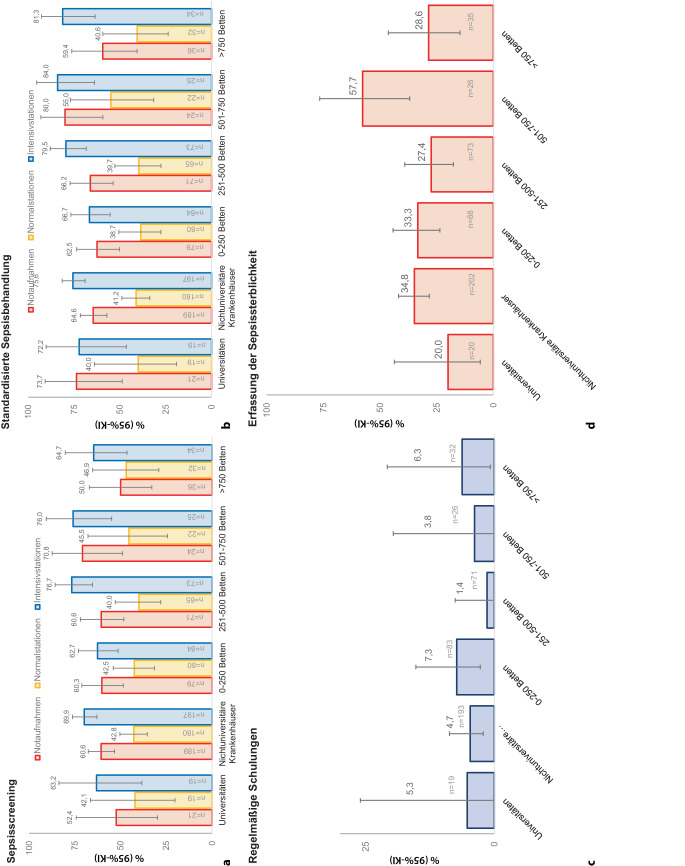
Sepsisscreening, standardisiertes Management, Schulungen und Outcome-Erfassung. **a** Sepsisscreening: Entsprechend IQTIG-Qualitätsindikator „Screening mittels Messinstrumenten zur Einstufung des Sepsisrisikos“. *Frage im ESCS:* *Haben Sie ein Protokoll oder standardisiertes Screening zur Erkennung einer Sepsis?* Die Frage wurde einzeln für die Bereiche Notaufnahme, Normalstation und Intensivstation gestellt. Dargestellt sind die prozentualen Anteile und 95 %-Konfidenzintervalle, in denen ein Sepsisscreening vorhanden war. **b** Standardisiertes Sepsismanagement: Entsprechend IQTIG-Qualitätsindikator „Arbeitsanweisung (SOP) zur Versorgung bei Sepsis.“ *Frage im ESCS: Haben Sie in Ihrem Krankenhaus ein Protokoll, Behandlungspfad oder Bundle zur Behandlung einer Sepsis?* Die Frage wurde einzeln für die Bereiche Notaufnahme, Normalstation und Intensivstation gestellt. Dargestellt sind die prozentualen Anteile und 95 %-Konfidenzintervalle, in denen eine standardisierte Sepsisbehandlung vorhanden war. **c** Regelmäßige Schulungen aller Bereiche: Entsprechend IQTIG-Qualitätsindikator „Regelmäßige Schulungen zu Erkennung, Risikoeinstufung und Therapie von Sepsis“ *Fragen im ESCS: Haben Sie ein Sepsistraining oder ein Programm zur Verbesserung der Sepsisqualität?* und *Sind regelmäßige Treffen und Trainings Bestandteil Ihres Sepsistrainings oder Sepsisqualitätsprogramms? (Aspekt der Regelmäßigkeit). ***d** Erfassung der Sepsissterblichkeit: Entsprechend IQTIG-Qualitätsindikator „Krankenhaus-Letalität bei Sepsis“ *Frage im ESCS: Bitte wählen Sie die Parameter aus, die in Ihrem Krankenhaus systematisch erfasst werden. Auswahl „Systematische Registrierung der Sepsismortalität“*

Ein standardisiertes Screening zur Erkennung einer Sepsis wurde am häufigsten von Intensivstationen (69,4 % (150/216) (95%-KI 62,8–75,5 %) berichtet. In Notaufnahmen war in 59,5 % (125/210) 59,5 % (95%-KI 52,6–66,2 %) und auf Normalstationen in 42,7 % (85/199) (95%-KI 35,7–49;9 %) ein Sepsisscreening etabliert (*p* < 0,05). Dabei bestanden zwischen universitären und nichtuniversitären Krankenhäusern keine signifikanten Unterschiede (*p* = 0,433). Ein Screening im gesamten Krankenhaus, d. h. Notaufnahme, Normalstation und Intensivstation), und nicht nur in Teilbereichen war in 34,8 % (95%-KI 27,9–42,3 %) etabliert. Die Raten des Sepsisscreenings in einzelnen Bereichen des Krankenhauses ist in Abb. 2 dargestellt. Wenn standardisierte Screeningprotokolle vorhanden waren, wurden diese teilweise nur bei Bedarf oder auf Anfrage und nicht systematisch bzw. täglich durchgeführt. Medical Emergency Teams (MET = Notfallteams oder intensivmedizinische Konsildienste, nicht aber Herzalarm- oder CPR-Teams) zur Evaluation potentiell kritisch kranker Patienten können zur Früherkennung septischer Patienten beitragen. Allerdings muss zur Auslösung solcher Teams bereits ein grundsätzliches Screening z. B. anhand von Vitalparametern erfolgt sein. Medical Emergency Teams waren in 50,4 % (123/244) der Krankenhäuser vorhanden, ohne signifikante Unterschiede zwischen kleinen und größeren Krankenhäusern (*p* = 0,225).

Ein standardisiertes Sepsismanagement anhand von Protokollen, Behandlungspfaden oder Bundles existierte vor allem in Notaufnahmen (65,2 % (129/198)) und Intensivstationen (75,0 % (159/212)) und deutlich weniger auf Normalstationen (41,1 % (78/190)) (*p* < 0,05) (Abb. 2). Ein krankenhausweites (d. h. in allen Bereichen vorhandenes) standardisiertes Management wurde von 36,6 % (95%-KI 29,2–44,5 %) der Krankenhäuser berichtet. Inhalte eines solch standardisierten Sepsismanagements waren vor allem die Bundle-Elemente der Surviving Sepsis Campaign (Lactatmessung, Blutkulturen vor Beginn einer antibiotischen Therapie, Flüssigkeitsgabe und der Einsatz von Vasopressoren bei hämodynamischer Instabilität), aber beispielsweise auch zusätzliche mikrobiologische Proben, CT- oder MRT-Bildgebung, eine Messung der Urinausscheidung, chirurgische Herdsanierung und ein erweitertes hämodynamisches Monitoring bzw. „passive leg raising“ zur Evaluierung eines Volumenbedarfes.

Schulungen zur Sepsis oder irgendeine Art des Trainings wurden von 19,8 % (42/212) (95%-KI 14,7–25,8 %) der Krankenhäuser berichtet. Solche Schulungen des ärztlichen und pflegerischen Dienstes, sowohl in der Notaufnahme, Normalstation als auch auf der Intensivstation, wurden von 8,0 % (17/212) (95%-KI 4,7–12,5 %) der Teilnehmer angegeben. Regelmäßige Schulungen des ärztlichen und pflegerischen Dienstes aller Abteilungen existierten in lediglich 4,7 % (10/212) (95%-KI 2,3–8,5 %) (Abb. 2). Nur 3,6 % (95%-KI 1,6–7,0 %) der Krankenhäuser unterstützten Sepsisschulungen, Weiterbildungen oder andere Sepsisaktivitäten finanziell.

Sepsisfälle wurden in 55,0 % (95%-KI 31,5–76,9 %) der universitären Krankenhäuser und 46,5 % (95%-KI 39,5–53,7 %) der nichtuniversitären Krankenhäuser systematisch erfasst (*p* = 0,469). Die Sepsissterblichkeit wurde in 33,3 % (74/222) der Krankenhäuser systematisch erfasst. In universitären Krankenhäusern war die Erfassung am seltensten (20 %, 4/20) und in Krankenhäusern mit 501 bis 750 Betten am häufigsten (57,7 %, 15/26). Zwischen den Krankenhäusern unterschiedlicher Bettengröße bestanden in Bezug auf die Erfassung der Sepsissterblichkeit signifikante Unterschiede (*p* = 0,037) (Abb. 2d).

### Mikrobiologische Diagnostik und antiinfektive Therapie

**Abb. 3:**
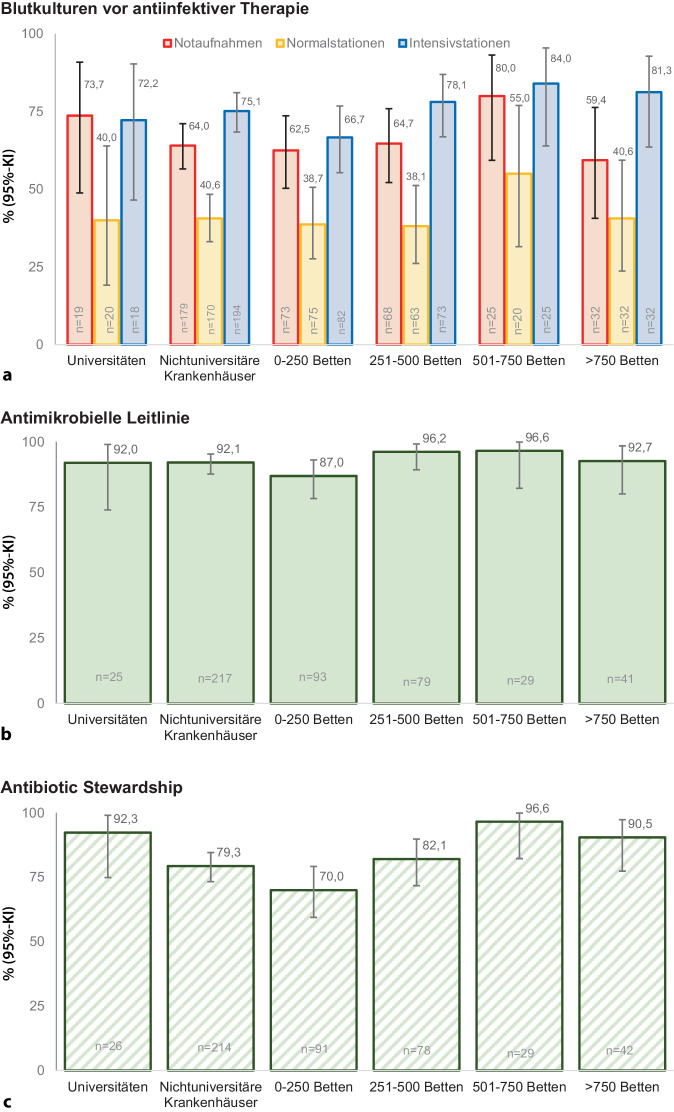
Abnahme von Blutkulturen, antimikrobielle Leitlinien und Antibiotic Stewardship. **a** Abnahme von Blutkulturen vor Beginn einer antiinfektiven Therapie. Entsprechend IQTIG-Qualitätsindikator: „Blutkulturen vor Beginn der antimikrobiellen Therapie der Sepsis“. *Frage im ESCS:* *Abnahme von Blutkulturen vor Beginn der antiinfektiven Therapie als Maßnahme in der Notaufnahme, auf der Normalstation und auf der Intensivstation. ***b** Antiinfektive Leitlinien für Patienten mit Sepsis und **c** Antibiotic Stewardship. Entsprechend IQTIG-Qualitätsindikator „Therapieleitlinie zur antiinfektiven Therapie unterstützt durch ein multidisziplinäres Antibiotic Stewardship-Team“. *Frage im ESCS: Haben Sie in Ihrem Krankenhaus eine SOP oder Leitlinie für die antimikrobielle Behandlung von Patienten mit Sepsis?* und *Haben Sie ein Antibiotic Stewardship Team (ABS) in Ihrem Krankenhaus?*

Die meisten Krankenhäuser (82,6 %, 194/235) verfügten über eine interne Leitlinie zur Abnahme von Blutkulturen. Blutkulturen vor Beginn einer antiinfektiven Therapie waren vor allem auf Intensivstation (74,5 % [95%-KI 68,1–80,2 %]) und in Notaufnahmen (64,6 % [95%-KI 57,6–71,3 %]) und seltener auf Normalstationen (40,5 % [95%-KI 33,5–47,9 %]) Bestandteil der diagnostischen Empfehlung. In Krankenhäusern mit einer Größe von 501 bis 750 Betten waren sie auf Intensivstationen mit 84,0 % (95%-KI 63,9–95,5 %) am häufigsten und auf Normalstationen in Krankenhäusern einer Größe von 251 bis 500 Betten mit 38,1 % (95%-KI 26,1–51,2 %) am seltensten Bestandteil der diagnostischen Empfehlung. Es bestanden keine Unterschiede zwischen den verschiedenen Krankenhausgrößen, jedoch zwischen Notaufnahmen, Normalstationen und Intensivstationen (*p* < 0,001) (Abb. 3).

Die mikrobiologischen Labore, in denen die Blutkulturen untersucht werden, waren meist nur eingeschränkt geöffnet. Eine ununterbrochene Rund-um-die-Uhr-Prozessierung von Blutkulturen, welche die Inkubation, die Erregeridentifikation und auch Ergebnisübermittlung beinhaltet (24/7 Service), wurde von 15,2 % (37/244) der Krankenhäuser berichtet. Krankenhäuser einer Bettengröße von 0 bis 750 Betten verfügten mit 12,9 % (26/202) seltener über einen mikrobiologischen 24/7 Service als Krankenhäuser mit mehr als 750 Betten mit 26,2 % (11/42) (*p* = 0,029).

Antimikrobielle Therapieleitlinien waren in 91,7 % (222/242) und Antibiotic Stewardship Teams in 80,8 % (194/240) der Krankenhäuser vorhanden (Abb. 3).

## Diskussion

Für Deutschland ist ein Qualitätssicherungsverfahren „Diagnostik, Therapie und Nachsorge der Sepsis“ (QS Sepsis) durch den Gemeinsamen Bundesausschuss beschlossen und ab dem 01.01.2026 umzusetzen [12]. Die darin enthaltenen Qualitätsindikatoren beziehen sich auf die Erkennung einer Sepsis, die Abnahme von Blutkulturen, Therapieleitlinien zur antiinfektiven Therapie, Antibiotic Stewardship Teams, Arbeitsanweisungen (SOP) zur Versorgung, regelmäßige Schulungen und die Erfassung des Outcomes [13–15]. Diese Indikatoren stellen sehr wesentliche Qualitätsaspekte dar, sind in ihrem Umfang aber stark reduziert und decken nicht alle Aspekte der Sepsisversorgung ab.

Informationen über die Implementierung standardisierter Maßnahmen zur Sepsisversorgung und Qualitätsindikatoren existieren bisher vorrangig in Rahmen von Studien [17, 18]. Für die Breite der Krankenhäuser in Deutschland existieren solche Informationen nicht. Im European Sepsis Care Survey, einer europaweiten Umfrage im Jahr 2021 und 2022 wurden Prozesse und Strukturen zur Sepsisversorgung erfasst [16]. Der Umfang des ESCS ging dabei deutlich über die im QS Sepsis erfassten Qualitätsindikatoren hinaus. So wurde zum Beispiel die Art und Weise des Sepsisscreenings, der Inhalt der standardisierten Therapie, aber auch das Vorhandensein von Infrastruktur zur Bildgebung und zur chirurgischen Herdsanierung und insbesondere die mikrobiologischen Blutkulturdiagnostik detailliert untersucht. In der Analyse wurden zudem Notaufnahmen, Normalstationen und Intensivstationen gesondert betrachtet [16]. Dies zeigt, dass die Qualitätsindikatoren des QS Sepsis die Sepsisversorgung keineswegs vollständig abbilden. Vielmehr sind sie der erste Schritt, um wichtige Schlüsselelemente in der Versorgung zu kennzeichnen und flächendeckend zu implementieren.

Mithilfe der Daten des ESCS konnten bereits vor Beginn des QS Sepsis Informationen zur Verfügbarkeit von Prozessen und Strukturen zur Sepsisversorgung in Deutschland untersucht werden.

Insgesamt konnten 253 Krankenhäusern in Deutschland in die Analyse eingeschlossen werden. Dies entspricht ca. 20 % aller akutmedizinischen Krankenhäuser in Deutschland [19]. In den untersuchten Krankenhäusern existierten Maßnahmen zur Sepsiserkennung im gesamten Krankenhaus nur in 34,8 % (95%-KI 27,9–42,3 %). Ein standardisiertes Management im Sinne von Therapieprotokollen oder Behandlungspfaden existierte krankenhausweit nur in 36,6 % (95%-KI 29,2–44,5 %). Regelmäßige, krankenhausweite Schulungen und Sepsistrainings für Ärzte und Pflegedienst wurden von 4,7 % (95%-KI 2,3–8,5 %) der Krankenhäuser berichtet.

In den USA wurde 2023 von Centers for Disease Control and Prevention (CDC) in Krankenhäusern ein Sepsisprogramm eingeführt, welches ebenfalls die Qualität der Sepsisversorgung verbessern soll [20, 21]. Kernelemente dieses Programms sind ebenfalls Maßnahmen zu Früherkennung und Behandlung der Sepsis sowie Schulungsmaßnahmen (Tab. 2). Im Jahr 2023 wurde über dieses US-Programm der erste Bericht veröffentlicht, der den Grad der Implementierung dieser Kernelemente in über 5000 US-Krankenhäusern beschreibt [22]. In diesem Bericht war ein Sepsisscreening in 69 % etabliert, 71 % verfügten über ein standardisiertes Management und in 72 % wurde eine Erfassung der Sterblichkeit als Outcome-Indikator durchgeführt. Auch bei der Durchführung von Schulungen (Pflegedienst 74 %, ärztlicher Dienst 55 %) wurden in den USA deutlich höhere Raten als in Deutschland erzielt [22, 23]. Der Einfluss von landesweiten Sepsisprogrammen und der Grad der Implementierung von Maßnahmen zu Früherkennung und Behandlung der Sepsis sollten in Zukunft mit vergleichbaren Outcome-Parametern (wie z. B. 30-Tage-Sterblichkeit) korreliert werden, idealerweise mit der Möglichkeit internationaler Vergleichbarkeit.

**Tab. 2:** Kernelemente des Krankenhaus-Sepsis-Programms in den USA

Kernelement	Beschreibung
Engagement der Krankenhausleitung	Unterstützung durch die Krankenhausleitung, um sicherzustellen, dass die Sepsisbekämpfung in den Krankenhäusern über die notwendigen personellen, finanziellen und informationstechnischen Ressourcen verfügt
Verantwortlichkeit	Ernennung von einem Leiter oder zwei Co-Leitern, die für die Programmziele und -ergebnisse verantwortlich sind
Multiprofessionelle Kompetenz	Einbindung wichtiger Partner im gesamten Krankenhaus und Gesundheitssystem zur Unterstützung der Sepsismaßnahmen und zur Qualitätsverbesserung
Handeln	Einführung von Strukturen und Verfahren zur Verbesserung der Erkennung und Behandlung von Sepsis (z. B. Krankenhausleitlinien, Behandlungspfade, Screeningprotokolle und Anordnungssätze)
Nachverfolgung	Messung der Epidemiologie, des Managements und der Ergebnisse, um die Auswirkungen von Sepsisinitiativen und die Fortschritte bei der Erreichung von Programmzielen zu bewerten
Berichterstattung	Bereitstellung von Daten zu Sepsismanagement und Ergebnissen für beteiligte Partner
Schulung	Aufklärung über Sepsis für Ärzte, Pflegepersonal, Patienten und Familienangehörige

Tabelle übersetzt aus: Prescott et al. JAMA [20, 21]

Im Gegensatz zum Qualitätssicherungsverfahren Sepsis in Deutschland sind im US-amerikanischen Programm unter anderem eine Unterstützung durch die Krankhausleitungen in Bezug auf ausreichende Ressourcen (personell und finanziell), die Benennung verantwortlicher Führungskräfte und die Einbindung einer multiprofessioneller Expertise ausdrücklich genannte Kernelemente [20, 21]. Fehlende Unterstützung der Krankenhausleitungen und mangelnde interdisziplinäre Kooperation waren neben nichtausreichenden personellen Ressourcen die Hauptursachen für das Scheitern von freiwilligen Sepsis Qualitätssicherungsprogrammen [17, 24–28]. Die Unterstützung durch Krankenhausleitungen und ein abteilungs- und berufsgruppenübergreifendes Engagement sind daher für den Erfolg eines Sepsisqualitätssicherungsverfahrens unerlässlich.

Die Abnahme von Blutkulturen vor Beginn einer antiinfektiven Therapie ist eine entscheidende Maßnahme für die Identifikation des Erregers, von Erregerlücken und Antibiotikaresistenzen [29]. Ergebnisse der Blutkulturdiagnostik ermöglichen eine Anpassung der Therapie im Verlauf im Sinne einer Eskalation, Deeskalation, wirksameren gezielten Therapie oder auch Beendigung der antiinfektiven Therapie [29–32].

Blutkulturen vor Beginn einer antiinfektiven Therapie waren in Notaufnahmen und auf Intensivstationen häufiger und auf Normalstationen signifikant seltener Teil der Diagnostik. Verbesserungspotential besteht hier aber auch im Bereich der Bearbeitung der Proben. Nur 15 % der Krankenhäuser verfügten nach der Umfrage über mikrobiologische Labore, welche rund um die Uhr eine Inkubation der Blutkulturen beginnen können, rund um die Uhr Erreger identifizieren können und rund um die Uhr Ergebnisse übermitteln. In einer europaweiten Umfrage über die mikrobiologische Diagnostik von Blutstrominfektionen (209 Labore, davon 11 Labore in Deutschland) wurde bereits 2019 über eingeschränkte Öffnungszeiten und Bearbeitung von Blutkulturen berichtet [33]. Eingeschränkte Bearbeitungszeiten in der mikrobiologischen Diagnostik können die Anpassung der Therapie auf eine zielgerichtete Therapie verzögern. Die Bedeutung einer schnellen mikrobiologischen Diagnostik bei Sepsis ist wesentlich [34–37]. Im Qualitätssicherungsverfahren Sepsis wird der Zeitpunkt der Blutkulturabnahme sowie des Eingangs des ersten mikrobiologischen Ergebnisses im Krankenhaus miterfasst. Damit wird es möglich, die Dauer der Bearbeitung der mikrobiologischen Analyse zu analysieren und daraus ggf. weitere Schritte abzuleiten [38].

Die vorliegende Studie zeigt einen großen Überblick über die aktuelle Sepsisversorgung in deutschen Krankenhäusern. Abgebildet wurde ca. ein Fünftel aller akutmedizinischen Krankenhäuser in Deutschland. Die ausgewerteten Informationen stammten zu 86 % direkt von Einrichtungsleitungen oder Oberärzten der Bereiche. Es muss aber berücksichtigt werden, dass die Ergebnisse anhand einer Befragung erhoben wurden und keine Validierung der Angaben vor Ort in den Krankenhäusern erfolgte [16]. Ein grundsätzliches Vorhandensein von Prozessen und Maßnahmen darf hier nicht mit einer grundsätzlich vorhandenen Umsetzung verwechselt werden. Gleichzeitig kann eine Behandlung aber auch ohne vorhandene *standard operating-procedures* (SOP) durch gut ausgebildetes Personal evidenzbasiert und auf hohem Niveau natürlich möglich sein.

Im Qualitätssicherungsverfahren Sepsis werden zukünftig, im Gegensatz zum ESCS, Qualitätsindikatoren fallbasiert überprüft. Dies bedeutet, dass die Anwendung von Maßnahmen am Einzelfall geprüft wird und nur das Vorhandensein einer SOP nicht ausreichen würde. Diese Studie kann daher nur den ersten Anhalt geben, bei welchen Qualitätsaspekten Verbesserungspotential bestehen könnte.

Das US-amerikanische Programm zeigt, dass Sepsisprogramme auf nationaler Ebene umsetzbar sind und diese Qualitätsindikatoren signifikant verbessern können [22, 23]. Diese Möglichkeit sollte auch in Deutschland genutzt werden. Gleichzeitig bedarf es aber auch ausreichender Ressourcen zur Umsetzung. Dies bedeutet mehr als nur die verpflichtende Etablierung und Überprüfung von Qualitätsindikatoren, nämlich ein sichtbares Engagement von Krankenhausleitungen, welche die notwendigen personellen und finanziellen Ressourcen sicherstellen und klare Verantwortlichkeiten schaffen. Innovative digitale Lösungen werden erforderlich sein, um Prozesse zu unterstützen, kontinuierlich zu erfassen und auszuwerten und dabei medizinisches Personal zu entlasten und nicht mehr zu belasten.

## Fazit für die Praxis

- Sepsis gehört zu den häufigsten Notfällen in Deutschland und ist mit einer hohen Sterblichkeit vergesellschaftet.
- Eine schnelle und zielgerichtete Therapie ist entscheidend, um die Krankheitsschwere zu reduzieren und die Sterblichkeit zu senken.
- Im Januar 2026 wurde das Qualitätssicherungsverfahrens „Diagnostik, Therapie und Nachsorge der Sepsis“ (QS Sepsis) durch den Gesetzgeber verpflichtend eingeführt.
- Die Ergebnisse des European Sepsis Care Survey zeigen ein erhebliches Verbesserungspotential im Bereich der Früherkennung (Sepsisscreening) und der standardisierten Sepsistherapie (Standardarbeitsanweisungen und Therapieempfehlungen in Krankenhäusern).
- Bisher sind regelmäßige Schulungen in allen Abteilungen des Krankenhauses (Notaufnahme, Normalstation und Intensivstation) in Deutschland sehr selten.
- Auch vor dem Hintergrund aktueller Leitlinienempfehlungen müssen Sepsisscreening, standardisierte Therapie und Schulungen von Krankenhausmitarbeitenden in Deutschland dringend verbessert werden.
- Im QS Sepsis werden diese Aspekte zukünftig adressiert. Dies könnte künftig dazu beitragen, das Behandlungsergebnis der Patienten zu verbessern.

## Supplementary Information

ESCS: Fragebogen.pdf

## Data Availability

Daten können in begründeten Fällen unter Wahrung des Datenschutzes zur Verfügung gestellt werden. Über die Weitergabe der Daten entscheidet der Lenkungsausschuss der Studie.
