# Supplementary material for: Sepsis care in Germany: quality indicators on diagnostics and treatment of sepsis
Source: Anaesthesiologie. 2026 Mar 19;75(5):333–43. [Article in German] doi: 10.1007/s00101-026-01663-5 (PMC13128695; doi:10.1007/s00101-026-01663-5)
Supplement: Supplementary file 1 — ESCS: Fragebogen.pdf [file 101_2026_1663_MOESM1_ESM.pdf]

Zusatzmaterial zum Beitrag „Sepsisversorgung in Deutschland: Qualitätsindikatoren zur Diagnostik und Therapie der Sepsis" von Christian S. Scheer, Evgeny A. Idelevich, Simon Oelsner et al. (2026) in *Die Anaesthesiologie.*

Beitrag und Zusatzmaterial stehen Ihnen auf www.springermedizin.de zur Verfügung. Bitte geben Sie dort den Beitragstitel in die Suche ein.

The EUROPEAN SEPSIS CARE SURVEY

Teilnehmer dieser Umfrage werden als Collaborators der European Sepsis Care Study Group geführt und PubMed gelistet.
Für Fragen senden Sie bitte eine Email an sepsissurvey@med.uni-greifswald.de

Die Umfrage erfasst potentiell vertrauliche Informationen wie z.B. bestimmte strukturelle Ausstattungen. Als Teilnehmer sollten Sie daher ggf. vorab eine Genehmigung zur Teilnahme einholen. Dies kann sich von Land zu Land unterscheiden. Die Entscheidung ob dies notwendig ist, liegt in der Verantwortung der teilnehmenden Person.

Die Umfrage erfragt nicht Ihren Namen. Es werden keine Patientendaten erfasst.
Rückschlüsse auf einzelne Krankenhäuser werden nicht möglich sein und nicht publiziert.

Alle erhobenen Daten werden vertraulich behandelt.

Impressum Datenschutzerklärung

Allgemeine Fragen

Bitte fragen Sie Kolleg*innen, wenn Sie die Fragen nicht beantworten können. Sie können die Umfrage auch mit Kolleg*innen teilen indem Sie den Link in Ihrem Browser weitergeben. Auf diese Art können Sie die Umfrage wechselseitig bearbeiten.

Bitte wählen Sie die Art Ihres Krankenhauses

- ○ Allgemeines Krankenhaus, Regelversorger
- ○ Universitätskrankenhaus
- ○ Privates Krankenhaus
- ○ Anderes (bitte angeben)

Wie viele Betten hat Ihr Krankenhaus?

Bitte geben Sie Ihre Position an

- ◯ Krankenhausdirektor
- ◯ Leiter einer Einrichtung (bitte Fachrichtung angeben) ________
- ◯ Facharzt (bitte Fachrichtung angeben) ________
- ◯ Andere (bitte angeben) ________

Impressum Datenschutzerklärung

Allgemeine Fragen

☐ Wie definieren Sie Sepsis in Ihrem Krankenhaus?

Mehrfachauswahl möglich

- ☐ Positive SIRS-Kriterien (systemisches inflammatorisches Response Syndrom) aufgrund einer Infektion.
- ☐ Neues Organversagen durch einen Anstieg des SOFA-Scores um ≥ 2 Punkte aufgrund einer Infektion.
- ☐ Andere (bitte beschreiben)
- ☐ Ich weiß nicht

Allgemeine Fragen

Welche Fachbereiche sind in Ihrem Krankenhaus vorhanden?

Mehrfachauswahl möglich.

- [ ] **Notaufnahme**
- [ ] **Intensivstation**
- [ ] **Allgemeinchirurgie**
- [ ] **Innere Medizin**
- [ ] Kardiologie
- [ ] Neurologie
- [ ] Unfallchirurgie
- [ ] Kinderheilkunde
- [ ] Gynäkologie
- [ ] Urologie
- [ ] Herzchirurgie
- [ ] Neurochirurgie
- [ ] Infektiologie
- [ ] **Klinische Labormedizin**
- [ ] **Mikrobiologisches Labor**
- [ ] **Externes mikrobiologisches Labor**
- [ ] **Radiologie**
- [ ] Krankenhaushygiene
- [ ] Andere (bitte aufführen)

Impressum Datenschutzerklärung

Allgemeine Fragen

Haben Sie in ihrem Krankenhaus ein Notfallteam bzw. intensivmedizinisches Konsilteam?

Notfallteam oder Konsilteam bezieht sich hier auf ein Team, welches zur Einschätzung potentiell kritisch kranker Patienten gerufen werden kann. Dies kann auch ein pflegegeführtes Team sein. Reanimationsteams oder Herzalarmteams sind nicht gemeint.

◯ Ja ◯ Nein ◯ Ich weiß nicht

Impressum Datenschutzerklärung

Allgemeine Fragen

Haben Sie in Ihrem Krankenhaus eine klinische Chemie/Labor?

- ◯ Ja, mit einer 24h Bereitschaft
- ◯ Ja, aber nur zu den Hauptarbeitszeiten
- ◯ Nein, wir verwenden eine externe klinische Chemie/Labor

Impressum Datenschutzerklärung

Allgemeine Fragen

☐ **Welche Analysemethoden stellt Ihr mikrobiologisches Labor zur Verfügung?**

	Montag bis Freitag zu den Hauptarbeitszeiten	Montag bis Freitag ist ein Notfalldienst auf Anfrage möglich	Montag bis Freitag 24h Dienst	Am Wochenende und an Ferientagen besteht ein eingeschränkter Dienst	Am Wochenende und an Ferientagen ist ein Notfalldienst auf Anfrage möglich	Am Wochenende und an Ferientagen 24h Dienst
Blutkulturinkubation	☐	☐	☐	☐	☐	☐
Grampräparat einer Blutkultur	☐	☐	☐	☐	☐	☐
Erregeridentifikation (z.B. MALDI_TOF)	☐	☐	☐	☐	☐	☐
Antibiogramm und Empfindlichkeitstestung	☐	☐	☐	☐	☐	☐
Mitteilung der Blutkulturergebnisse	☐	☐	☐	☐	☐	☐

Impressum Datenschutzerklärung

Allgemeine Fragen

☐ Haben Sie in Ihrem Krankenhaus eine mikrobiologische Schnelltestung verfügbar?

Mehrfachauswahl möglich

- ☐ Ja, wir haben eine Schnelldiagnostik aus positiven Blutkulturen (z.B. PCR, direkt MALDI-TOF, MALDI-TOF nach Kurzinkubation)
- ☐ Ja, wir haben eine antimikrobielle Empfindlichkeitstestung direkt aus positiven Blutkulturen.
- ☐ Nein
- ☐ Ich weiß nicht

Impressum Datenschutzerklärung

Allgemeine Fragen

Sind Sie mit den angebotenen diagnostischen Möglichkeiten Ihres mikrobiologischen Labors für Sepsispatienten zufrieden?

Bitte auswählen

Impressum Datenschutzerklärung

Allgemeine Fragen

Welche Verbesserungen in der mikrobiologischen Diagnostik wären wünschenswert?

Mehrfachauswahl möglich

- ☐ Die mikrobiologische Diagnostik der Sepsis sollte schneller sein
- ☐ Die mikrobiologische Diagnostik der Sepsis sollte präziser sein
- ☐ Genauere therapeutische Ratschläge (Auswahl des Antibiotikums, Dosierung, Dauer)
- ☐ mikrobiologische Bereitschaft 24/7
- ☐ Keine, ich bin zufrieden

Impressum Datenschutzerklärung

Allgemeine Fragen

☐ Welche Untersuchungsmöglichkeiten sind in Ihrer Radiologie verfügbar?

CT = Computertomografie; MR = Magnetresonanztomografie

- ☐ CT / MR 24h verfügbar
- ☐ CT / MR sind nur zu den Hauptarbeitszeiten möglich
- ☐ Patienten müssen für eine Computertomographie zu einer externen Radiologie transportiert werden
- ☐ Kein CT / MR verfügbar (z.B. nur Röntgen-Thorax)
- ☐ Interventionelle Radiologie

Impressum Datenschutzerklärung

Allgemeine Fragen

Haben Sie in Ihrem Krankenhaus eine SOP oder Leitlinie für die antimikrobielle Behandlung von Patienten mit Sepsis?

- ◯ Ja
- ◯ Nein
- ◯ Ich weiß nicht

Impressum Datenschutzerklärung

Allgemeine Fragen

Haben Sie eine Leitlinie oder ein Protokoll für die Blutkulturentnahme?

- Ja
- Nein
- Ich weiß nicht

Impressum Datenschutzerklärung

Allgemeine Fragen

Wie verfolgen Sie den Status Ihrer Blutkulturen von der Abnahme über die Inkubation bis zum Endergebnis?

- ◯ Wir haben kein derartiges System
- ◯ Der Status der Blutkulturen kann mit Verzögerung in unserem Labor- oder Krankenhausinformationssystem eingesehen werden.
- ◯ Der Status der Blutkulturen kann in Echtzeit in unserem Labor- oder Krankenhausinformationssystem eingesehen werden.
- ◯ Andere (bitte beschreiben)
- ◯ Ich weiß nicht

Impressum Datenschutzerklärung

Allgemeine Fragen

Haben Sie ein Antibiotic Stewardship Team (ABS) in Ihrem Krankenhaus?

- ◯ Ja
- ◯ Nein
- ◯ Ich weiß nicht

Impressum Datenschutzerklärung

Allgemeine Fragen

Welche Aussagen können Sie in Hinblick auf die Herdsanierung treffen?

Mehrfachauswahl möglich

- ☐ Die operative Herdsanierung ist in unserem Krankenhaus 24/7 verfügbar
- ☐ Die operative Herdsanierung ist nur zu den Hauptarbeitszeiten (tagsüber) verfügbar
- ☐ Die interventionelle Herdsanierung durch die radiologische Abteilung ist in unserem Krankenhaus 24/7 verfügbar
- ☐ Die interventionelle Herdsanierung durch die radiologische Abteilung ist nur zu den Hauptarbeitszeiten (tagsüber)verfügbar
- ☐ Eine Herdsanierung ist in unserem Krankenhaus nicht möglich, der Patient muss verlegt werden
- ☐ Ich weiß nicht

Impressum Datenschutzerklärung

Allgemeine Fragen

Wie priorisieren Sie die Herdsanierung im Vergleich zur antiinfektiven Therapie bei septischen Patienten?

- ◯ Die Herdsanierung wird vor einer Antibiotikatherapie priorisiert.
- ◯ Wenn Patienten Antibiotika erhalten ist die Herdsanierung von nachgeordneter Bedeutung
- ◯ Herdsanierung und antiinfektive Therapie sind von gleichrangiger Bedeutung
- ◯ Ich weiß nicht

Impressum Datenschutzerklärung

Fragen zur Sepsisversorgung in der Notaufnahme

Wie viele Patienten behandeln Sie üblicherweise pro Monat in Ihrer Notaufnahme?

[ ] Patienten / Monat

Bitte schätzen Sie die Sepsisfälle in Ihrer Notaufnahme pro Monat.

[ ] Sepsisfälle / Monat

Benutzen Sie ein Triage-System oder Score in Ihrer Notaufnahme?

- ○ Ja
- ○ Nein
- ○ Ich weiß nicht

Bitte benennen Sie die Triage-Systeme und Scores, die in Ihrer Notaufnahme verwendet werden

Mehrfachauswahl möglich

- ☐ Manchester Triage System (MTS)
- ☐ Emergency Severity Index
- ☐ ABCDE-Schema
- ☐ Frühwarn-Scores (z.B. NEWS, MEWS)
- ☐ RETTS (Rapid Emergency Triage and Treatment System)
- ☐ STaRT (Simple Triage and rapid treatment)
- ☐ mSTaRT
- ☐ BASIC (Bleeding, Airway, Shock, Immobilisation after Classification)
- ☐ SOFA
- ☐ qSOFA
- ☐ Andere (bitte beschreiben) ____________
- ☐ Keine

Haben Sie ein Protokoll oder standardisiertes Screening zur Erkennung von Sepsis in Ihrer Notaufnahme?

- ○ Ja
- ○ Nein
- ○ Ich weiß nicht

Bitte wählen Sie die Kriterien aus, die zum Sepsisscreening in Ihrer

Notaufnahme verwendet werden.

Mehrfachauswahl möglich - bitte alle zutreffenden Kriterien auswählen

- ☐ Atemfrequenz
- ☐ Temperatur
- ☐ Mentale Veränderungen (z.B. Glasgow Coma Scale)
- ☐ Verhaltensauffälligkeiten
- ☐ Herzfrequenz
- ☐ Blutdruck
- ☐ Urinausscheidung
- ☐ Laktat
- ☐ Organdysfunktionen (Mentalstatus, kardiovaskuläres System, Respiration, Nierenfunktion)
- ☐ qSOFA
- ☐ SOFA
- ☐ SIRS-Kriterien
- ☐ Frühwarn Scores (z.B. NEWS, MEWS)
- ☐ weiße Blutkörperchen / Leukozyten
- ☐ Procalcitonin (PCT)
- ☐ C-reaktives Protein (CRP)
- ☐ IL-6
- ☐ vermutete Infektion
- ☐ bestätigte Infektion
- ☐ [illegible]

- [ ] Andere (bitte benennen)

Haben Sie ein Protokoll, Behandlungspfad oder Bundle zur Behandlung einer Sepsis in Ihrer Notaufnahme?

- ○ Ja
- ○ Nein
- ○ Ich weiß nicht

Bitte wählen Sie die Maßnahmen, die im Sepsisprotokoll, Behandlungspfad oder Bundle Ihrer Notaufnahme enthalten sind.

Mehrfachauswahl möglich

- [ ] Bestimmung des Laktatwertes
- [ ] Abnahme von Blutkulturen vor Beginn der antiinfektiven Therapie
- [ ] Gabe von Breitspektrum-Antibiotika
- [ ] Zügige Infusion von 30ml/kg Kristalloide bei Hypotension oder Serum-Laktat ≥ 4 mmol/l
- [ ] Gabe von Vasopressoren bei persistierender Hypotonie unter Volumentherapie um einen MAP ≥ 65 mmHg zu erreichen

Existiert ein bestimmter zeitlicher Rahmen für die Durchführung dieser Maßnahmen?

- ◯ Nein
- ◯ 1h
- ◯ 2h
- ◯ 6h
- ◯ 12h
- ◯ 24h

Bitte wählen Sie weitere Maßnahmen, die in Ihrer Notaufnahme durchgeführt werden und den dazugehörigen Zeitrahmen

Mehrfachauswahl möglich

					Zeitrahmen			
	Teil des Sepsis-Bundle	Teil des Sepsisprotokolls oder Behandlungspfades	Nur auf Anforderung	Nie	innerhalb von 1-6 Stunden	innerhalb von 12 Stunden	innerhalb von 24 Stunden	kein Zeitrahmen
Körperliche Untersuchung	☐	☐	☐	☐	◯	◯	◯	◯
Laboruntersuchung (Leukozyten, Gerinnung, Nierenfunktion, Leberfunktion)	☐	☐	☐	☐	◯	◯	◯	◯
Erhebung des SOFA Scores	☐	☐	☐	☐	◯	◯	◯	◯
CT zur Identifikation des Infektionsfokus	☐	☐	☐	☐	◯	◯	◯	◯

Ultraschall zur Identifikation des Infektionsfokus	☐	☐	☐	☐	○	○	○	○
Röntgen Thorax zur Identifikation des Infektionsfokus	☐	☐	☐	☐	○	○	○	○
Mikrobiologische Proben (z.B. Urin, Abstriche, Liquor)	☐	☐	☐	☐	○	○	○	○
Katheterisierung /Bestimmung der Urinausscheidung	☐	☐	☐	☐	○	○	○	○
Schnelle Herdsanierung (chirurgisch oder interventionell)	☐	☐	☐	☐	○	○	○	○
Anlage eines zentralvenösen Katheters (ZVK)	☐	☐	☐	☐	○	○	○	○
Bestimmung von ScvO2	☐	☐	☐	☐	○	○	○	○
Arterielle Blutdruckmessung	☐	☐	☐	☐	○	○	○	○
Lagerungsmanöver (passive leg raising) oder Flüssigkeitsbolus (fluid challange) zur Bestimmung der Volumenreagibilität	☐	☐	☐	☐	○	○	○	○
Messung von Herzminutenvolumen, Schlagvolumen, Pulsdruck	☐	☐	☐	☐	○	○	○	○
Reguläre Konsultation								

durch einen Infektiologen	☐	☐	☐	☐	○	○	○	○
Procalcitonin-gesteuerte antiinfektive Therapie	☐	☐	☐	☐	○	○	○	○
MRSA-PCR Testung	☐	☐	☐	☐	○	○	○	○
Andere mikrobiologische Schnelltestung (bitte beschreiben)	☐	☐	☐	☐	○	○	○	○
Andere Maßnahmen (bitte beschreiben)	☐	☐	☐	☐	○	○	○	○

Haben Sie die Möglichkeit einer Blutgas- und Laktatbestimmung in Ihrer Notaufnahme?

○ Ja ○ Nein ○ Ich weiß nicht

Wann nehmen Sie Blutkulturen in Ihrer Notaufnahme ab?

Mehrfachauswahl möglich

- ☐ Sie werden bei Fieber abgenommen
- ☐ Sie werden bei Vorliegen eines Schocks abgenommen
- ☐ Sie werden bei Verdacht auf eine Infektion abgenommen
- ☐ Sie werden nicht routinemäßig abgenommen
- ☐ Andere (bitte benennen)

Wer nimmt die Blutkulturen in Ihrer Notaufnahme ab?

Mehrfachauswahl möglich

- ☐ Pflegepersonal
- ☐ Ärztliches Personal
- ☐ Spezialisiertes Personal für Venenpunktionen
- ☐ Abhängig von der Verfügbarkeit des Personals

Welchen Stelle/Ort bevorzugen Sie zur Blutkulturentnahme in Ihrer Notaufnahme?

	bevorzugt	erste Alternative	zweite Alternative	dritte Alternative	nie verwendet
Direkte Venenpunktion an zwei verschiedenen Stellen	○	○	○	○	○
Direkte Venenpunktion an einer Stelle	○	○	○	○	○
Neue (bis 24h) Katheter (arteriell oder zentralvenös)	○	○	○	○	○
Alte (>24h) Katheter (arteriell oder zentralvenös)	○	○	○	○	○
Alte Katheter bei Verdacht auf eine Katheterinfektion	○	○	○	○	○

Wie viele Blutkulturen nehmen Sie bei Sepsisverdacht in Ihrer Notaufnahme ab?

Ein Set besteht aus 2 Flaschen, eine aerobe Flasche und eine anaerobe Flasche

	immer, 100% der Blutkulturentnahmen	in mehr als 75% der Blutkulturentnahmen	in 50-75% der Blutkulturentnahmen	in 25-50% der Blutkulturentnahmen	in weniger als 25% der Blutkulturentnahmen	nie (0%) der Blutkulturentnahmen
1 Set (2 Flaschen)	○	○	○	○	○	○
2 Sets (4 Flaschen)	○	○	○	○	○	○
3 Sets (6 Flaschen)	○	○	○	○	○	○
4 Sets (8 Flaschen)	○	○	○	○	○	○
mehr als 4 Sets	○	○	○	○	○	○

Mit welcher Priorität werden Blutkulturen in Ihrer Notaufnahme verschickt?

- Sie werden unverzüglich nach Abnahme weggeschickt
- Sie werden zu festen Zeiten (z. B. Morgenschicht, Nachmittagsschicht, Nachtschicht) und unabhängig von der Probenahmezeit verschickt.
- Sie werden von der abnehmenden Station/Einrichtung gelagert und durch das Laborpersonal eingesammelt, sobald sie verfügbar sind.
- Ich weiß nicht

Innerhalb von wie vielen Stunden/Tagen nach Blutkulturentnahme erhalten Sie ein erstes Ergebnis (z.B. mikroskopischer Befund) einer positiven Blutkultur die in Ihrer Notaufnahme abgenommen wurde.

Bitte schätzen Sie, falls keine exakten Daten verfügbar sind.

- 0-12h
- 12-24h (1 Tag)
- 24-48h (1-2 Tage)
- 48-72h (2-3 Tage)
- 72-96h (3-4 Tage)
- mehr als 4 Tage
- Ich kenne die Daten dazu nicht und kann keine Schätzung abgeben

Wer kann in Ihrer Notaufnahme Antibiotika verordnen und welche?

Mehrfachauswahl möglich

- [ ] Ärzte können jede Art von Antibiotika verordnen
- [ ] Ärzte können nur vorrätige Antibiotika verordnen. Keine Sonderrezepte
- [ ] Ärzte benötigen eine Freigabe durch die Pharmakologie bei der Verordnung bestimmter Antibiotika
- [ ] Ärzte benötigen eine Freigabe durch einen Facharzt
- [ ] Ärzte müssen die Infektiologie konsultieren

Impressum Datenschutzerklärung

Fragen zur Sepsisversorgung auf der Normalstation und/ oder Intermediate Care Unit (nicht Intensivstation)

Bitte wählen Sie die Art der Normalstation für die Sie eine Aussage machen möchten, eine Mehrfachauswahl ist möglich.

Die Antworten gelten nur für gewählte Stationsarten. Bitte schreiben Sie eine Email an sepsissurvey@med.uni-greifswald.de, wenn Sie Antworten für weitere oder unterschiedliche Stationen geben möchten. Sie erhalten dann einen Zusatzfragebogen.

- ☐ Chirurgische Stationen
- ☐ Internistische Stationen
- ☐ Intermediate Care Stationen (ohne Beatmung)
- ☐ Andere (bitte beschreiben)

Wie viele Betten haben Ihre Stationen im Durchschnitt?

Betten

Wie viele Patienten behandeln Sie üblicherweise pro Monat auf einer Normalstation durchschnittlicher Größe?

Patienten / Monat

Bitte schätzen Sie die Sepsisfälle pro Monat auf einer Normalstation durchschnittlicher Größe.

[ ] Sepsisfälle / Monat

Haben Sie ein Protokoll oder standardisiertes Screening zur Erkennung einer Sepsis auf der Normalstation?

- ◯ Ja
- ◯ Nein
- ◯ Ich weiß nicht

Wie oft werden Patienten auf der peripheren Station in Hinblick auf eine Sepsis gescreent?

- ◯ Täglich
- ◯ Nur bei Bedarf
- ◯ Andere (bitte benennen) [ ]
- ◯ Ich weiß nicht

Bitte wählen Sie die Kritrien die zum Sepsisscreening auf der Normalstation verwendet werden.

Mehrfachauswahl möglich - bitte alle zutreffenden Kriterien auswählen

- [ ] Atemfrequenz
- [ ] Temperatur
- [ ] Herzfrequenz

- [ ] Weiße Blutkörperchen / Leukozyten
- [ ] Mentale Veränderungen (z.B. Glasgow Coma Scale)
- [ ] Verhaltensänderungen
- [ ] Blutdruck
- [ ] Urinausscheidung
- [ ] Laktat
- [ ] Organdysfunktionen (Mentalstatus, kardiovaskuläres System, Respiration, Nierenfunktion)
- [ ] qSOFA
- [ ] SOFA
- [ ] SIRS-Kriterien
- [ ] Frühwarn-Scores (z.B. NEWS, MEWS)
- [ ] Procalcitonin (PCT)
- [ ] C-reaktives Protein (CRP)
- [ ] IL-6
- [ ] vermutete Infektion
- [ ] bestätigte Infektion
- [ ] Andere (bitte benennen)

Haben Sie ein Protokoll, Behandlungspfad oder Bundle zur Behandlung einer Sepsis auf der Normalstation?

- ◯ Ja
- ◯ Nein
- ◯ Ich weiß nicht

Bitte wählen Sie die Maßnahmen, die im Sepsisprotokoll, Behandlungspfad oder Bundle Ihrer Normalstation enthalten sind.

Mehrfachauswahl möglich

- ☐ Messung des Laktatwertes
- ☐ Abnahme von Blutkulturen vor Beginn der antiinfektiven Therapie
- ☐ Gabe von Breitspektrum-Antibiotika
- ☐ Zügige Infusion von 30ml/kg Kristalloide bei Hypotension oder Serum-Laktat ≥ 4 mmol/l
- ☐ Gabe von Vasopressoren bei persistierender Hypotonie unter Volumentherapie um einen MAP ≥ 65 mmHg zu erreichen

Existiert ein bestimmter zeitlicher Rahmen für die Durchführung dieser Maßnahmen?

- ◯ Nein
- ◯ 1h
- ◯ 2h
- ◯ 6h
- ◯ 12h
- ◯ 24h

Bitte wählen Sie weitere Maßnahmen, die auf der Normalstation durchgeführt werden und den dazugehörigen Zeitrahmen

Mehrfachauswahl möglich.

					Zeitrahmen			
	Teil des Sepsis-Bundle	Teil des Sepsisprotokolls oder Behandlungspfades	Nur auf Anforderung	Nie	innerhalb von 1-6 Stunden	innerhalb von 12 Stunden	innerhalb von 24 Stunden	kein Zeitrahmen
Körperliche Untersuchung	☐	☐	☐	☐	◯	◯	◯	◯
Laboruntersuchung (Leukozyten, Gerinnung, Nierenfunktion, Leberfunktion)	☐	☐	☐	☐	◯	◯	◯	◯
Erhebung des SOFA Scores	☐	☐	☐	☐	◯	◯	◯	◯
CT zur Identifikation des Infektionsfokus	☐	☐	☐	☐	◯	◯	◯	◯

Ultraschall zur Identifikation des Infektionsfokus	☐	☐	☐	☐	○	○	○	○
Röntgen Thorax zur Identifikation des Infektionsfokus	☐	☐	☐	☐	○	○	○	○
Mikrobiologische Proben (z.B. Urin, Abstriche, Liquor)	☐	☐	☐	☐	○	○	○	○
Katheterisierung /Bestimmung der Urinausscheidung	☐	☐	☐	☐	○	○	○	○
Schnelle Herdsanierung (chirurgisch oder interventionell)	☐	☐	☐	☐	○	○	○	○
Anlage eines zentralvenösen Katheters (ZVK)	☐	☐	☐	☐	○	○	○	○
Bestimmung von ScvO2	☐	☐	☐	☐	○	○	○	○
Invasive arterielle Blutdruckmessung	☐	☐	☐	☐	○	○	○	○
Lagerungsmanöver (passive leg raising) oder Flüssigkeitsbolus (fluid challange) zur Bestimmung der Volumenreagibilität	☐	☐	☐	☐	○	○	○	○
Messung von Herzminutenvolumen, Schlagvolumen, Pulsdruck	☐	☐	☐	☐	○	○	○	○
Reguläre Konsultation								

durch einen Infektiologen	☐	☐	☐	☐	○	○	○	○
Procalcitonin-gesteuerte antiinfektive Therapie	☐	☐	☐	☐	○	○	○	○
MRSA-PCR Testung	☐	☐	☐	☐	○	○	○	○
Andere mikrobiologische Schnelltestung (bitte beschreiben) ______	☐	☐	☐	☐	○	○	○	○
Andere Maßnahmen (bitte beschreiben) ______	☐	☐	☐	☐	○	○	○	○

Haben Sie die Möglichkeit einer Blutgas- und Laktatbestimmung auf Ihrer Normalstation?

- ○ Ja
- ○ Nein
- ○ Ich weiß nicht

Werden Patienten mit einer Sepsis auf eine Intermediate Care Station oder Intensivstation verlegt?

- ○ Ja, wir verlegen alle Sepsis-Patienten
- ○ Ja, aber es werden nur Patienten mit höherem Schweregrad (septischem Schock oder Beatmung) verlegt
- ○ Nein, wir verlegen Patienten mit Sepsis / septischen Schock nicht
- ○ Ich weiß nicht

Wer behandelt Sepsispatienten auf der Normalstation?

Mehrfachauswahl möglich

- ☐ Stationsarzt
- ☐ Facharzt für Infektionskrankheiten
- ☐ Antibiotic Stewardship Team
- ☐ Andere (bitte benennen) ______

Wer kann auf der Normalstation Antibiotika verordnen und welche?

Mehrfachauswahl möglich

- ☐ Ärzte können jede Art von Antibiotika verordnen
- ☐ Ärzte können nur vorrätige Antibiotika verordnen. Keine Sonderrezepte
- ☐ Ärzte benötigen eine Freigabe durch die Pharmakologie bei der Verordnung bestimmter Antibiotika
- ☐ Ärzte benötigen eine Freigabe durch einen Facharzt
- ☐ Ärzte müssen die Infektiologie konsultieren

Erhalten Sie Ratschläge bezüglich einer geeigneten antiinfektiven Therapie auf der Normalstation?

- ○ Ja
- ○ Nein
- ○ Ich weiß nicht

Wer berät Sie auf der peripheren Station bezüglich der antiinfektiven Therapien?

Mehrfachauswahl möglich

- ☐ Antibiotic Stewardship Team
- ☐ Infektiologie
- ☐ Mikrobiologie
- ☐ Andere (bitte benennen) ______

Wann nehmen Sie auf der Normalstation Blutkulturen ab?

Mehrfachauswahl möglich

- ☐ Sie werden bei Fieber abgenommen
- ☐ Sie werden bei Vorliegen eines Schocks abgenommen
- ☐ Sie werden in jedem Fall bei Vorliegen oder dem Verdacht auf eine Infektion abgenommen
- ☐ Sie werden nicht routinemäßig abgenommen

Wer nimmt die Blutkulturen auf der Normalstation ab?

Mehrfachauswahl möglich

- ☐ Pflegepersonal
- ☐ ärztliches Personal
- ☐ Spezialisiertes Personal für Venenpunktion
- ☐ Abhängig von der Verfügbarkeit des Personals

Welchen Stelle/Ort bevorzugen Sie zur Blutkulturentnahme auf der Normalstation?

	bevorzugt	erste Alternative	zweite Alternative	dritte Alternative	nie verwendet
Direkte Venenpunktion an zwei verschiedenen Stellen	○	○	○	○	○
Direkte Venenpunktion an einer Stelle	○	○	○	○	○
Neue (bis 24h) Katheter (arteriell oder zentralvenös)	○	○	○	○	○
Alte (>24h) Katheter (arteriell oder zentralvenös)	○	○	○	○	○
Alte(>24h) Katheter bei Verdacht auf eine Katheterinfektion	○	○	○	○	○

Wie viele Blutkulturen nehmen Sie bei Sepsisverdacht auf der Normalstation ab?

Ein Set besteht aus 2 Flaschen, eine aerobe und eine anaerobe Flasche

	immer, 100% der Blutkulturentnahmen	in mehr als 75% der Blutkulturentnahmen	in 50-75% der Blutkulturentnahmen	in 25-50% der Blutkulturentnahmen	in weniger als 25% der Blutkulturentnahmen	nie (0%) der Blutkulturentnahmen
1 Set (2 Flaschen)	○	○	○	○	○	○
2 Sets (4 Flaschen)	○	○	○	○	○	○
3 Sets (6 Flaschen)	○	○	○	○	○	○
4 Sets (8 Flaschen)	○	○	○	○	○	○
mehr als 4 Sets	○	○	○	○	○	○

Mit welcher Priorität werden Blutkulturen, die auf der Normalstation abgenommen wurden, in das Labor geschickt?

- ◯ Sie werden unverzüglich nach Abnahme weggeschickt
- ◯ Sie werden zu festen Zeiten (z. B. Morgenschicht, Nachmittagsschicht, Nachtschicht) und unabhängig von der Probenahmezeit verschickt.
- ◯ Sie werden von der abnehmenden Station/Einrichtung gelagert und durch das Laborpersonal eingesammelt, sobald sie verfügbar sind
- ◯ Ich weiß nicht

Innerhalb von wie vielen Stunden/Tagen nach Blutkulturentnahme erhalten Sie ein erstes(vorläufiges) Ergebnis (z.B. mikroskopischer Befund) einer positiven Blutkultur, die auf der Normalstation abgenommen wurde.

Bitte schätzen Sie, falls keine exakten Daten verfügbar sind

- ◯ 0-12h
- ◯ 12-24h (1 Tag)
- ◯ 24-48h (1-2 Tage)
- ◯ 48-72h (2-3 Tage)
- ◯ 72-96h (3-4 Tage)
- ◯ mehr als 4 Tage
- ◯ Ich kenne die Daten dazu nicht und kann keine Schätzung abgeben

Wie lange dauert es üblicherweise, bis Sie auf der Normalstation das endgültige mikrobiologische Ergebnis (Erregeridentifikation und Resistogramm) erhalten?

Bitte schätzen Sie, falls keine exakten Daten verfügbar sind

- 1 Tag
- 2 Tage
- 3 Tage
- 4 Tage
- 5 Tage
- 6 Tage
- 7 Tage
- länger als 7 Tage

Wie erhalten Sie Informationen über die ersten (vorläufigen) Ergebnisse (z.B. mikroskopischer Befund, Erregeridentifikation) einer positiven Blutkultur, die auf der Normalstation abgenommen wurde?

Mehrfachauswahl möglich

- [ ] Ich muss nachsehen und erhalte die Informationen als Papierbefund / Fax
- [ ] Ich muss nachsehen und erhalte die Informationen über das klinikinterne IT-System
- [ ] Das Labor meldet sich bei mir direkt
- [ ] Ich muss im Labor telefonisch nachfragen
- [ ] Die Informationen wird an anderes Personal (z.B. Pflegepersonal) weitergegeben
- [ ] Andere (bitte benennen)

Impressum Datenschutzerklärung

Fragen zur Sepsisversorgung auf der Intensivstation

☐ Bitte wählen Sie die Art der Intensivstation für die Sie eine Aussage machen möchten, eine Mehrfachauswahl ist möglich.

Die Antworten gelten nur für gewählte Stationsarten. Bitte schreiben Sie eine Email an sepsissurvey@med.uni-greifswald.de, wenn Sie Antworten für weitere oder unterschiedliche Stationen geben möchten. Sie erhalten dann einen Zusatzfragebogen.

- ☐ Interdisziplinär
- ☐ Chirurgisch
- ☐ Internistisch
- ☐ Anästhesiologisch
- ☐ Andere (bitte benennen)

☐ Welche Maßnahmen sind auf Ihrer Intensivstation verfügbar?

Mehrfachauswahl möglich.

- ☐ Beatmung
- ☐ Blutgasanalyse /Laktatmessung
- ☐ Nierenersatztherapie
- ☐ Hämodynamisches Monitoring
- ☐ ECMO

Wie viele Intensivbetten hat Ihr Krankenhaus?

Betten [ ]

Wie viele Patienten behandeln Sie üblicherweise pro Monat auf der Intensivstation?

[ ] Patienten / Monat

Bitte schätzen Sie die Anzahl der Sepsisfälle, die Sie pro Monat auf der Intensivstation behandeln.

[ ] Sepsisfälle / Monat

Sind bei Ihnen spezielle Protokolle oder ein standardisiertes Screening zur Erkennung der Sepsis verfügbar?

○ Ja ○ Nein ○ Ich weiß nicht

Wie oft werden Patienten auf der Intensivstation auf eine Sepsis gescreent?

- [ ] Täglich
- [ ] Nur bei Bedarf
- [ ] Andere (bitte benennen) [ ]
- [ ] Ich weiß nicht

Bitte wählen Sie die Kriterien aus, die auf Ihrer Intensivstation zur

Identifikation einer Sepsis verwendet werden.

Mehrfachauswahl möglich

- [ ] Atemfrequenz
- [ ] Temperatur
- [ ] Mentale Veränderung (z.B. Glasgow Coma Scale)
- [ ] Verhaltensänderung
- [ ] Herzfrequenz
- [ ] Blutdruck
- [ ] Urinausscheidung
- [ ] Laktat
- [ ] Organdysfunktionen (Mentalstatus, kardiovaskuläres System, Respiration, Nierenfunktion)
- [ ] qSOFA
- [ ] SOFA Score
- [ ] SIRS-Kriterien
- [ ] Frühwarn-Score (z.B. NEWS, MEWS)
- [ ] Weiße Blutkörperchen / Leukozyten
- [ ] Procalcitonin
- [ ] C-reaktives Protein (CRP)
- [ ] IL-6
- [ ] vermutete Infektion
- [ ] bestätigte Infektion
- [ ] [illegible]

☐ Andere (bitte benennen)

Haben Sie ein Sepsisprotokoll, Behandlungspfad oder Bundle zur Sepsisbehandlung auf der Intensivstation?

○ Ja ○ Nein ○ Ich weiß nicht

Bitte wählen Sie die Maßnahmen, die in Ihrem Sepsis-Bundle enthalten sind.

Mehrfachauswahl möglich

☐ Messung des Laktatwertes

☐ Abnahme von Blutkulturen vor Beginn einer antiinfektiven Therapie

☐ Gabe von Breitspektrum-Antibiotika

☐ Zügige Infusion von 30ml/kg Kristalloide bei Hypotension oder Serum-Laktat ≥ 4mmol/l

☐ Gabe von Vasopressoren bei persistierender Hypotonie unter Volumentherapieum einen MAP ≥ 65 mmHg zu erreichen

Existiert ein bestimmter zeitlicher Rahmen für die Durchführung dieser Maßnahmen?

- ◯ Nein
- ◯ 1h
- ◯ 2h
- ◯ 6h
- ◯ 12h
- ◯ 24h

Bitte wählen Sie weitere Maßnahmen, die auf der Intensivstation durchgeführt werden und den dazugehörigen Zeitrahmen

Mehrfachauswahl möglich.

					Zeitrahmen			
	Teil des Sepsis-Bundle	Teil des Sepsisprotokolls oder Behandlungspfades	Nur auf Anforderung	Nie	innerhalb von 1-6 Stunden	innerhalb von 12 Stunden	innerhalb von 24 Stunden	kein Zeitrahmen
Körperliche Untersuchung	☐	☐	☐	☐	◯	◯	◯	◯
Laboruntersuchung (Leukozyten, Gerinnung, Nierenfunktion, Leberfunktion)	☐	☐	☐	☐	◯	◯	◯	◯
Erhebung des SOFA Scores	☐	☐	☐	☐	◯	◯	◯	◯
CT zur Identifikation des Infektionsfokus	☐	☐	☐	☐	◯	◯	◯	◯

Ultraschall zur Identifikation des Infektionsfokus	☐	☐	☐	☐	◯	◯	◯	◯
Röntgen-Thorax zur Identifikation des Infektionsfokus	☐	☐	☐	☐	◯	◯	◯	◯
Mikrobiologische Proben (z.B. Urin, Abstriche, Liquor)	☐	☐	☐	☐	◯	◯	◯	◯
Katheterisierung /Bestimmung der Urinausscheidung	☐	☐	☐	☐	◯	◯	◯	◯
Schnelle Herdsanierung (chirurgisch oder interventionell)	☐	☐	☐	☐	◯	◯	◯	◯
Anlage eines zentralvenösen Katheters (ZVK)	☐	☐	☐	☐	◯	◯	◯	◯
Bestimmung ScvO2	☐	☐	☐	☐	◯	◯	◯	◯
Invasive arterielle Blutdruckmessung	☐	☐	☐	☐	◯	◯	◯	◯
Lagerungsmanöver (passive leg raising) oder Flüssigkeitsbolus (fluid challange) zur Bestimmung der Volumenreagibilität	☐	☐	☐	☐	◯	◯	◯	◯
Messung von Herzminutenvolumen, Schlagvolumen, Pulsdruck	☐	☐	☐	☐	◯	◯	◯	◯
Reguläre Konsultation durch einen	☐	☐	☐	☐	◯	◯	◯	◯

Infektiologen

Procalcitonin-gesteuerte antiinfektive Therapie ☐ ☐ ☐ ☐ ○ ○ ○ ○

MRSA-PCR Testung ☐ ☐ ☐ ☐ ○ ○ ○ ○

Andere mikrobiologische Untersuchungen (bitte benennen) ☐ ☐ ☐ ☐ ○ ○ ○ ○

Andere Maßnahmen (bitte benennen) ☐ ☐ ☐ ☐ ○ ○ ○ ○

Wer kann auf der Intensivstation Antibiotika verordnen und welche?

Mehrfachauswahl möglich

- ☐ Ärzte können jede Art von Antibiotika verordnen
- ☐ Ärzte können nur gelistete Antibiotika verordnen. Keine Sonderrezepte
- ☐ Ärzte benötigen eine Freigabe durch die Pharmakologie bei der Verordnung bestimmter Antibiotika
- ☐ Ärzte benötigen eine Freigabe durch einen Facharzt
- ☐ Ärzte müssen die Infektiologie konsultieren

Bekommen Sie auf der Intensivstation Ratschläge bezüglich der antiinfektiven Therapie?

- ◯ Ja
- ◯ Nein
- ◯ Ich weiß nicht

Wer berät Sie auf der Intensivstation bezüglich der antiinfektiven Therapien?

- ☐ Antibiotic Stewardship Team
- ☐ Infektiologie
- ☐ Mikrobiologie
- ☐ Andere (bitte benennen) ______

Wie wird der Volumenbedarf bei Patienten im septischen Schock überwacht?

Mehrfachauswahl möglich

- [ ] Blutdruck / Herzfrequenz
- [ ] Urinausscheidung
- [ ] ZVD, ScvO2
- [ ] Ultraschall von Thorax und Herz
- [ ] Pulsdruckvariation, Herzminutenvolumen, Schlagvolumen
- [ ] Laktat Clearance / Normalisierung
- [ ] Ansprechen auf einen Volumenbolus oder passives Anheben der Beine
- [ ] Wir verwenden keine der aufgeführten Maßnahmen

Welcher der Vasopressoren wird bevorzugt eingesetzt?

- ○ Adrenalin
- ○ Noradrenalin
- ○ Vasopressin
- ○ Andere (bitte benennen)

Welche Infusionslösungen werden für Patienten mit Sepsis oder septischen Schock verwendet?

Mehrfachauswahl möglich

- ☐ Isotone Kochsalzlösung (NaCl 0,9%)
- ☐ Balancierte Kristalloide
- ☐ Hydroxyethylstärke (HES)
- ☐ Albumin
- ☐ Gelatine
- ☐ Dextran
- ☐ Andere (bitte benennen)

Wann nehmen Sie Blutkulturen auf der Intensivstation ab?

Mehrfachauswahl möglich

- ☐ Sie werden bei Fieber abgenommen
- ☐ Sie werden bei Vorliegen eines Schocks abgenommen
- ☐ Sie werden in jedem Fall bei Vorliegen oder dem Verdacht auf eine Infektion abgenommen
- ☐ Sie werden nicht routinemäßig abgenommen

Wer nimmt die Blutkulturen auf der Intensivstation ab?

Mehrfachauswahl möglich.

- ☐ Pflegepersonal
- ☐ Ärztliches Personal
- ☐ Spezialisiertes Personal für Venenpunktionen
- ☐ Abhängig von der Verfügbarkeit des Personals

Welchen Stelle/Ort bevorzugen Sie zur Blutkulturentnahme auf der Intensivstation?

	bevorzugt	erste Alternative	zweite Alternative	dritte Alternative	nie verwendet
Direkte Venenpunktion an zwei verschiedenen Stellen	○	○	○	○	○
Direkte Venenpunktion an einer Stelle	○	○	○	○	○
Neue (bis 24h) Katheter	○	○	○	○	○
Alte (>24h) Katheter (arteriell oder zentralvenös)	○	○	○	○	○
Alte Katheter bei Verdacht auf eine Katheterinfektion	○	○	○	○	○

Wie viele Blutkulturen nehmen Sie bei Sepsisverdacht auf der Intensivstation ab?

1 Set besteht aus 2 Flaschen, eine aerobe und eine anaerobe Flasche

	immer, 100% der Blutkulturentnahmen	in mehr als 75% der Blutkulturentnahmen	in 50-75% der Blutkulturentnahmen	in 25-50% der Blutkulturentnahmen	in weniger als 25% der Blutkulturentnahmen	nie, 0% der Blutkulturentnahmen
1 Set (2 Flaschen)	○	○	○	○	○	○
2 Sets (4 Flaschen)	○	○	○	○	○	○
3 Sets (6 Flaschen)	○	○	○	○	○	○
4 Sets (8 Flaschen)	○	○	○	○	○	○
mehr als 4 Sets	○	○	○	○	○	○

Mit welcher Priorität werden Blutkulturen, die auf der Intensivstation abgenommen wurden, in das Labor geschickt?

- ○ Sie werden unverzüglich nach Abnahme weggeschickt
- ○ Sie werden zu festen Zeiten (z. B. Morgenschicht, Nachmittagsschicht, Nachtschicht) und unabhängig von der Probenahmezeit verschickt.
- ○ Sie werden von der abnehmenden Station/Einrichtung gelagert und durch das Laborpersonal eingesammelt, sobald sie verfügbar sind
- ○ Ich weiß nicht

Innerhalb von wie vielen Stunden/Tagen nach Blutkulturentnahme erhalten Sie ein erstes (vorläufiges) Ergebnis (z.B. mikroskopischer Befund) einer positiven Blutkultur, die auf der Intensivstation abgenommen wurde.

Bitte schätzen Sie, falls keine exakten Daten verfügbar sind

- ◯ 0-12h
- ◯ 12-24h (1 Tag)
- ◯ 24-48h (2 Tage)
- ◯ 48-72h (2-3 Tage)
- ◯ 72-96h (3-4 Tage)
- ◯ mehr als 4 Tage
- ◯ Ich kenne die Daten dazu nicht und kann keine Schätzung abgeben

Wie lange dauert es üblicherweise, bis Sie das endgültige mikrobiologische Ergebnis (Erregeridentifikation und Resistogramm) einer Blutkultur erhalten, welche auf der Intensivstation abgenommen wurde?

Bitte schätzen Sie, falls keine exakten Daten verfügbar sind

- ◯ 1 Tag
- ◯ 2 Tage
- ◯ 3 Tage
- ◯ 4 Tage
- ◯ 5 Tage
- ◯ 6 Tage
- ◯ 7 Tage
- ◯ länger als 7 Tage

Wie erhalten Sie Informationen über die ersten (vorläufigen) Ergebnisse (z.B. mikroskopischer Befund, Erregeridentifikation) einer positiven Blutkultur, die auf der Intensivstation abgenommen wurde?

Mehrfachauswahl möglich

- [ ] Ich muss nachsehen und erhalte die Informationen als Papierbefund / Fax
- [ ] Ich muss nachsehen und erhalte die Informationen über das klinikinterne IT-System
- [ ] Das Labor meldet sich bei mir direkt
- [ ] Ich muss im Labor telefonisch nachfragen
- [ ] Die Information wird an anderes Personal (z.B. Pflegepersonal) weitergegeben
- [ ] Andere (bitte benennen)

Impressum Datenschutzerklärung

Qualitätsmanagement

Haben Sie ein Sepsistraining oder ein Programm zur Verbesserung der Sepsisqualität?

◯ Ja ◯ Nein ◯ Ich weiß nicht

Wer ist in das Sepsistraining / Sepsisqualitätsprogramm einbezogen?

	Notaufnahme	periphere Station	Intensivstatio	Ambulanzen	Andere Bereiche
Pflegepersonal der ...	☐	☐	☐	☐	☐
Ärztliches Personal der ...	☐	☐	☐	☐	☐
Andere (z.B. Physiotherapie - bitte benennen) ______	☐	☐	☐	☐	☐

Bitte wählen Sie die Bestandteile Ihres Sepsistrainings oder Sepsisqualitätsprogramme aus.

Mehrfachauswahl möglich

- [ ] Regelmäßige Treffen und Trainings
- [ ] Fallbesprechungen mit dem Team und Feedback
- [ ] Informationsmaterial (z.B. Taschenkarten, Poster)
- [ ] Externe Peer-Reviews
- [ ] Andere (bitte benennen)

Bitte wählen Sie die Parameter aus, die in Ihrem Krankenhaus systematisch erfasst werden.

Mehrfachauswahl möglich

- ☐ Systematische Registrierung der Sepsisfälle
- ☐ Systematische Registrierung der Sepsismortalität
- ☐ Messung der "time-to-antibiotics" / Zeit bis zur Antibiotikagabe
- ☐ Erfassung der Umsetzungscompliance des Sepsis-Bundle
- ☐ Schwere der Sepsisfälle
- ☐ Anzahl der Blutkulturen
- ☐ Andere (bitte benennen)
- ☐ Wir erheben diese Parameter nicht

Investieren Sie zusätzliches Geld in Programme zur Bekämpfung der Sepsis oder spezialisiertes Sepsis-Personal?

- ○ Ja (Bitte benennen Sie für was das Geld genutzt wird)
- ○ Nein

Werden Qualitätsdaten zu Kontaminationsraten von Blutkulturen in Ihrem Krankenhaus erfasst?

- ◯ Ja, (bitte benennen Sie die Blutkulturkontaminationsrate in % bezogen auf das letzte Jahr) ______
- ◯ Nein
- ◯ Ich weiß nicht

Hat COVID-19 die Sepsisbehandlung in Ihrem Bereich beeinflusst?

- ◯ Nein
- ◯ Ja (bitte benennen) ______

Impressum Datenschutzerklärung
